# Trastuzumab deruxtecan versus treatment of physician’s choice in previously treated Asian patients with HER2-low unresectable/metastatic breast cancer: subgroup analysis of the DESTINY-Breast04 study

**DOI:** 10.1007/s12282-024-01600-7

**Published:** 2024-06-17

**Authors:** Toshinari Yamashita, Joo Hyuk Sohn, Eriko Tokunaga, Naoki Niikura, Yeon Hee Park, Keun Seok Lee, Yee Soo Chae, Binghe Xu, Xiaojia Wang, Seock-Ah Im, Wei Li, Yen-Shen Lu, Cecilia Orbegoso Aguilar, Soichiro Nishijima, Yuji Nishiyama, Masahiro Sugihara, Shanu Modi, Junji Tsurutani

**Affiliations:** 1 Kanagawa Cancer Center, Kanagawa, Japan; 2 Yonsei University Health System, Seoul, Republic of Korea; 3 NHO Kyushu Cancer Center, Fukuoka, Japan; 4 Tokai University School of Medicine Hospital, Kanagawa, Japan; 5 Samsung Medical Center, Seoul, Republic of Korea; 6 National Cancer Center, Gyeonggi-do, Republic of Korea; 7 Kyungpook National University Chilgok Hospital, Daegu, Republic of Korea; 8 Cancer Hospital Chinese Academy of Medical Sciences, Peking Union Medical College, Beijing, China; 9 Zhejiang Cancer Hospital, Hangzhou, China; 10 Seoul National University Hospital, Cancer Research Institute, Seoul National University College of Medicine, Seoul, Republic of Korea; 11 The First Hospital of Jilin University, Jilin, China; 12 National Taiwan University Hospital, Taipei, Taiwan; 13 Daiichi Sankyo France SAS, Rueil-Malmaison, France; 14 Daiichi Sankyo, Co., Ltd, Tokyo, Japan; 15 Memorial Sloan Kettering Cancer Center, New York, NY USA; 16 The Innovative Center of Translational Research and Clinical Science for Cancer Therapy, Showa University Hospital, Tokyo, Japan; 17 Advanced Cancer Translational Research Institute, Showa University, Tokyo, Japan

**Keywords:** Advanced breast cancer, Asia, HER2-low, Interstitial lung disease, Trastuzumab deruxtecan

## Abstract

**Background:**

In the global phase 3 DESTINY-Breast04 study (NCT03734029), the anti-human epidermal growth factor 2 (HER2) antibody–drug conjugate trastuzumab deruxtecan (T-DXd) demonstrated a statistically significant improvement in progression-free survival (PFS) and overall survival (OS), with manageable safety compared with treatment of physician’s choice (TPC) in patients with HER2-low metastatic breast cancer (mBC) who had received 1–2 prior lines of chemotherapy.

**Methods:**

This subgroup analysis examined the efficacy and safety of T-DXd versus TPC in 213 patients from Asian countries and regions who were enrolled in the DESTINY-Breast04 trial and randomized to T-DXd (n = 147) or TPC (n = 66).

**Results:**

Median PFS with T-DXd and TPC was 10.9 and 5.3 months, respectively, in Asian patients with hormone receptor-positive mBC, and 10.9 and 4.6 months, respectively, in the overall Asian population. In both populations, median OS was not reached with T-DXd and was 19.9 months with TPC. The objective response rate was higher with T-DXd versus TPC in all Asian patients. Median treatment duration was 8.4 months with T-DXd and 3.5 months with TPC. The most common grade ≥ 3 drug-related treatment-emergent adverse events in Asian patients treated with T-DXd were neutropenia (16.3%), anemia (12.9%), and leukopenia (11.6%); the incidences of neutropenia and leukopenia were higher with TPC versus T-DXd. Adjudicated drug-related interstitial lung disease or pneumonitis with T-DXd was 14.3%; the majority of events were grade 1–2.

**Conclusions:**

T-DXd demonstrated clinically meaningful survival benefits versus TPC in Asian HER2-low mBC patients, regardless of hormone receptor status, with no new safety signals.

**Clinical trial registration number:**

ClinicalTrials.gov, NCT03734029.

**Supplementary Information:**

The online version contains supplementary material available at 10.1007/s12282-024-01600-7.

## Introduction

Breast cancer (BC) is the most commonly diagnosed cancer among women worldwide and is the fifth leading cause of cancer-related deaths [1]. In fact, approximately 60% of patients with a human epidermal growth factor 2 (HER2)-negative BC diagnosis have tumors with low HER2 expression (HER2-low status), defined as an immunohistochemistry (IHC) score of 1 + or 2 + and a negative in situ hybridization (ISH) score [2–4]. Despite the significant advancements in treatment options for patients with BC, historically, those with HER2-low metastatic BC (mBC) had limited targeted treatment options in the late-line setting [3, 5]. However, HER2-low mBC can now be treated with the new anti-HER2 antibody–drug conjugates (ADCs), namely trastuzumab deruxtecan (T-DXd) [6].

T-DXd has been approved for use in patients with HER2-positive and HER2-low mBC by the United States Food and Drug Administration and the European Medicines Agency [6, 7]. It is also approved for both these indications in some regions in Asia (i.e., China, Japan, and Taiwan) [8–10]. According to the Pan-Asian adapted European Society for Medical Oncology (ESMO) guidelines, T-DXd can be considered for patients with hormone receptor-positive or hormone receptor-negative, HER2-low unresectable and/or mBC previously treated with one or two prior lines of chemotherapy [11]. The National Comprehensive Cancer Network also prefers T-DXd as a second-line treatment option in patients with hormone receptor-positive, HER2-low, IHC 1 + or 2 + /ISH-negative mBC [12].

Phase 1 and 2 studies of T-DXd have shown promising results in heavily pretreated patients with HER2-low mBC, with an objective response rate (ORR) of 37.0–37.5% and progression-free survival (PFS) of 6.3–11.1 months [13–15]. The global phase 3 DESTINY-Breast04 study was designed to evaluate the efficacy and safety of T-DXd compared with treatment of physician’s choice (TPC) in patients with HER2-low mBC who had previously been treated with chemotherapy [16]. Over a median follow-up of 18 months, T-DXd demonstrated superior efficacy with regard to PFS (primary endpoint) in patients with hormone receptor-positive HER2-low mBC compared with TPC (median PFS 10.1 vs 5.4 months, respectively; hazard ratio [HR] 0.51; p < 0.001). The study also met the key secondary endpoints of PFS among all patients (median PFS 9.9 vs 5.1 months, respectively; HR 0.50; p < 0.001), as well as overall survival (OS) in patients with hormone receptor-positive disease (23.9 vs 17.5 months, respectively; HR 0.64; p = 0.003) and all patients (23.4 vs 16.8 months, respectively; HR 0.64; p = 0.001). Although no new safety signals for T-DXd were observed in this population, the incidence of low-grade drug-related interstitial lung disease (ILD) or pneumonitis was higher with T-DXd versus TPC [16]. With a longer term treatment duration (median follow-up 32 months), preliminary data suggested that T-DXd provides a sustained clinically meaningful improvement in PFS and OS compared with TPC in patients with HER2-low mBC, regardless of hormone receptor status, with an overall safety profile that was similar to that reported in the primary analysis [17].

The toxicity profile with T-DXd might vary among ethnic groups, and its therapeutic index is of great interest in specific populations. Although T-DXd is currently standard of care for patients with HER2-low mBC in Asian countries and regions, there are limited data available on its efficacy and safety in Asian patients. Therefore, a prespecified subgroup analysis was conducted to examine the efficacy and safety of T-DXd versus TPC in Asian patients with HER2-low mBC who took part in the DESTINY-Breast04 study.

## Materials and methods

### Study design and participants

The design of the open-label, multicenter, randomized, active-controlled, phase 3 DESTINY-Breast04 study (ClinicalTrials.gov identifier: NCT03734029) has been described previously [16]. Briefly, adults (aged ≥ 18 years) with pathologically documented HER2-low unresectable and/or mBC were enrolled if they had previously received one or two prior lines of chemotherapy for metastatic disease or had progressed within 6 months of adjuvant chemotherapy. Patients were randomized (2:1) to receive T-DXd 5.4 mg/kg intravenously every 3 weeks or TPC (i.e., capecitabine, eribulin, gemcitabine, paclitaxel, or nab-paclitaxel), in accordance with the drug label, until the withdrawal of consent, unacceptable toxicity, or progressive disease (PD). Key exclusion criteria included previous treatment with anti-HER2 therapy; history of noninfectious ILD or pneumonitis that required corticosteroids, or suspected ILD on imaging at screening; and spinal cord compression or brain metastases that were symptomatic or required treatment.

The study was conducted in accordance with the ethical standards of the Declaration of Helsinki, the International Council for Harmonization Good Clinical Practice guidelines and other local regulations, and was approved by the Institutional Review Board at each study site. Written informed consent was collected from all patients before study initiation.

### Outcomes

The primary endpoint was PFS in patients with hormone receptor-positive disease, as determined by a blinded independent central review (BICR). Key secondary endpoints included PFS among all patients and OS in the hormone receptor-positive cohort and among all patients.

Other secondary endpoints included PFS (as determined by investigator) and ORR (i.e., the proportion of patients with a best overall response of complete response [CR] or partial response [PR]), disease control rate (DCR), clinical benefit rate (CBR; i.e., sum of CR, PR and > 6 months’ stable disease rates), and the duration of response and time to response (as determined by BICR and investigator).

Safety assessments included treatment-emergent adverse events (TEAEs) and drug-related TEAEs, identified using the Medical Dictionary for Regulatory Activities, version 25.0, and graded according to the National Cancer Institute Common Terminology Criteria for Adverse Events, version 5.0. An independent adjudication committee evaluated all potential cases of ILD or pneumonitis.

### Statistical analysis

This pre-planned subgroup analysis included data from patients enrolled from Asian countries and regions in the DESTINY-Breast04 study. Efficacy analyses were performed in the full analysis set, which included all randomized patients. Safety analyses were conducted in patients who received at least one dose of study treatment.

Baseline characteristics, response rates, and safety were assessed using descriptive statistics. Continuous data were summarized for each treatment group using median and range (minimum and maximum). Categorical data were summarized using the number and percentage of patients.

The survival distribution of PFS and OS was estimated using the Kaplan–Meier method, and two-sided 95% confidence intervals (CIs) of quartile event times were calculated using the Brookmeyer and Crowley method. HRs estimates for PFS and OS, and corresponding two-sided 95% CIs were determined using the Cox proportional hazards regression model.

Statistical analyses were performed using SAS version 9.4.

## Results

### Patients

Between 27 December 2018 and 31 December 2021, 557 patients with HER2-low mBC were randomized into the DESTINY-Breast04 study. Of these, 213 patients (38.2%) were from Asian countries or regions (85 patients from Japan, 62 from China, 57 from the Republic of Korea, and nine from Taiwan). The data reported in this paper are exclusively from these Asian patients.

Among the patients, 147 were randomized to T-DXd (128/147 [87.1%] were hormone receptor-positive), and 66 were randomized to TPC (60/66 [90.9%] were hormone receptor-positive; Online Resource 1). Sixty-three patients received at least one dose of chemotherapy in the TPC group; 36 patients (57.1%) received eribulin, 12 (19.0%) received capecitabine, seven (11.1%) received gemcitabine, six (9.5%) received nab-paclitaxel, and two (3.2%) received paclitaxel.

No major differences in patient baseline demographics and clinical characteristics were observed between treatment groups among hormone receptor-positive Asian patients and all Asian patients (Table 1). Across all treatment groups, patients had a median age of 55.3–56.8 years, 55.0–58.6% of patients had an ECOG PS score of 0, and 59.1–63.3% of patients had a HER2-low IHC score of 1 + . The most common metastatic site was the liver (59.1–72.7% of patients), and 89.4–100% of patients had received prior endocrine therapy and chemotherapy.

**Table 1:** Baseline characteristics of Asian patients included in the DESTINY-Breast04 study

	Hormone receptor-positive Asian patients		All Asian patients	
	T-DXd n = 128	TPC n = 60	T-DXd n = 147	TPC n = 66
Age, median (range), years	56.8 (31.5–79.1)	55.3 (28.4–80.0)	56.6 (31.5–79.1)	55.3 (28.4–80.0)
Sex, n (%)				
Male	0	0	0	0
Female	128 (100)	60 (100)	147 (100)	66 (100)
Region, n (%)				
Republic of Korea	33 (25.8)	18 (30.0)	38 (25.9)	19 (28.8)
Japan	47 (36.7)	25 (41.7)	56 (38.1)	29 (43.9)
China	42 (32.8)	16 (26.7)	45 (30.6)	17 (25.8)
Taiwan	6 (4.7)	1 (1.7)	8 (5.4)	1 (1.5)
HER2-low status,^a^ n (%)				
IHC 1 +	81 (63.3)	37 (61.7)	91 (61.9)	39 (59.1)
IHC 2 + and ISH-negative	47 (36.7)	23 (38.3)	56 (38.1)	27 (40.9)
ECOG PS, n (%)				
0	75 (58.6)	33 (55.0)	81 (55.1)	37 (56.1)
1	53 (41.4)	27 (45.0)	66 (44.9)	29 (43.9)
Hormone receptor, n (%)				
Positive with prior CDK4/6 inhibitor	66 (51.6)	30 (50.0)	66 (44.9)	30 (45.5)
Positive without prior CDK4/6 inhibitor	62 (48.4)	30 (50.0)	62 (42.2)	30 (45.5)
Negative	0	0	19 (12.9)	6 (9.1)
Metastasis, n (%)				
Brain	11 (8.6)	2 (3.3)	15 (10.2)	3 (4.5)
Liver	93 (72.7)	38 (63.3)	100 (68.0)	39 (59.1)
Lung	42 (32.8)	23 (38.3)	53 (36.1)	24 (36.4)
Renal function,^b^ n (%)				
Normal	65 (50.8)	27 (45.0)	75 (51.0)	28 (42.4)
Mild impairment	48 (37.5)	24 (40.0)	56 (38.1)	27 (40.9)
Moderate impairment	11 (8.6)	6 (10.0)	12 (8.2)	8 (12.1)
Severe impairment	0	0	0	0
End-stage renal disease	0	0	0	0
Missing data	4 (3.1)	3 (5.0)	4 (2.7)	3 (4.5)
Lines of therapy for metastatic disease				
Median (range)	3 (1–8)	3 (1–8)	3 (1–8)	3 (1–8)
Number of lines, n (%)				
1	8 (6.3)	6 (10.0)	17 (11.6)	9 (13.6)
2	35 (27.3)	14 (23.3)	40 (27.2)	17 (25.8)
≥ 3	85 (66.4)	40 (66.7)	90 (61.2)	40 (60.6)
Previous cancer therapy in any setting, n (%)				
Targeted therapy	84 (65.6)	41 (68.3)	90 (61.2)	43 (65.2)
CDK4/6 inhibitor	66 (51.6)	30 (50.0)	67 (45.6)	30 (45.5)
Immunotherapy	2 (1.6)	2 (3.3)	3 (2.0)	4 (6.1)
Other	49 (38.3)	25 (41.7)	54 (36.7)	26 (39.4)
Endocrine therapy	127 (99.2)	59 (98.3)	135 (91.8)	59 (89.4)
Chemotherapy	128 (100)	60 (100)	147 (100)	66 (100)

*CDK4/6* cyclin-dependent kinases 4 and 6, *CrCl* creatinine clearance, *ECOG PS* ECOG performance status, *HER2* human epidermal growth factor receptor 2, *IHC* immunohistochemistry, *ISH* in situ hybridization, *T-DXd* trastuzumab deruxtecan, *TPC* treatment of physician’s choice

^a^Low expression of HER2 was defined as a score of 1 + on IHC analysis or as an IHC score of 2 + and negative results on in situ hybridization

^b^CrCl ≥ 90 mL/min = normal, CrCl ≥ 60 to < 90 mL/min = mild impairment, CrCl ≥ 30 to < 60 mL/min = moderate impairment, CrCl ≥ 15 to < 30 mL/min = severe impairment, and CrCl < 15 mL/min = end-stage renal disease

At data cut-off (January 11, 2022), treatment was ongoing in 20 patients (13.6%) in the T-DXd group and in one patient (1.6%) in the TPC group (Online Resource 1). The primary reasons for treatment discontinuation in the T-DXd and TPC groups were PD (58.5% and 85.7%, respectively), adverse events (17.7% and 6.3%, respectively), and patient withdrawal (5.4% and 4.8%, respectively). The median study duration was 16.8 and 15.4 months in the T-DXd and TPC groups, respectively.

### Efficacy

**Fig. 1:**
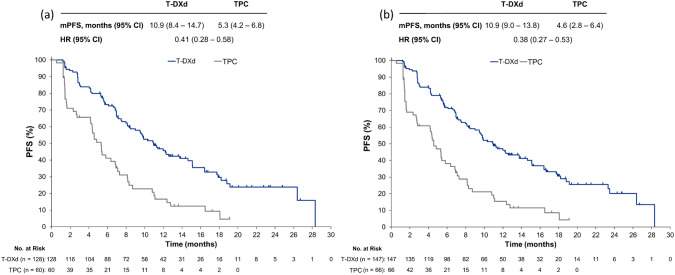
Progression-free survival by blinded independent central review in **a** the hormone receptor-positive Asian cohort, and **b** all Asian patients (hormone receptor-positive and -negative). *CI* confidence interval, *HR* hazard ratio, *mPFS* median progression-free survival, *T-DXd* trastuzumab deruxtecan, *TPC* treatment of physician’s choice

In the hormone receptor-positive cohort, the median PFS as determined by BICR was 10.9 (95% CI 8.4–14.7) months with T-DXd and 5.3 (95% CI 4.2–6.8) months with TPC, corresponding to an HR of 0.41 (95% CI 0.28–0.58) in favor of T-DXd (Fig. 1a). A similar trend was observed in the overall population; median PFS was 10.9 (95% CI 9.0–13.8) months in the T-DXd group and 4.6 (95% CI 2.8–6.4) months in the TPC group (HR 0.38; 95% CI 0.27–0.53; Fig. 1b).

**Fig. 2:**
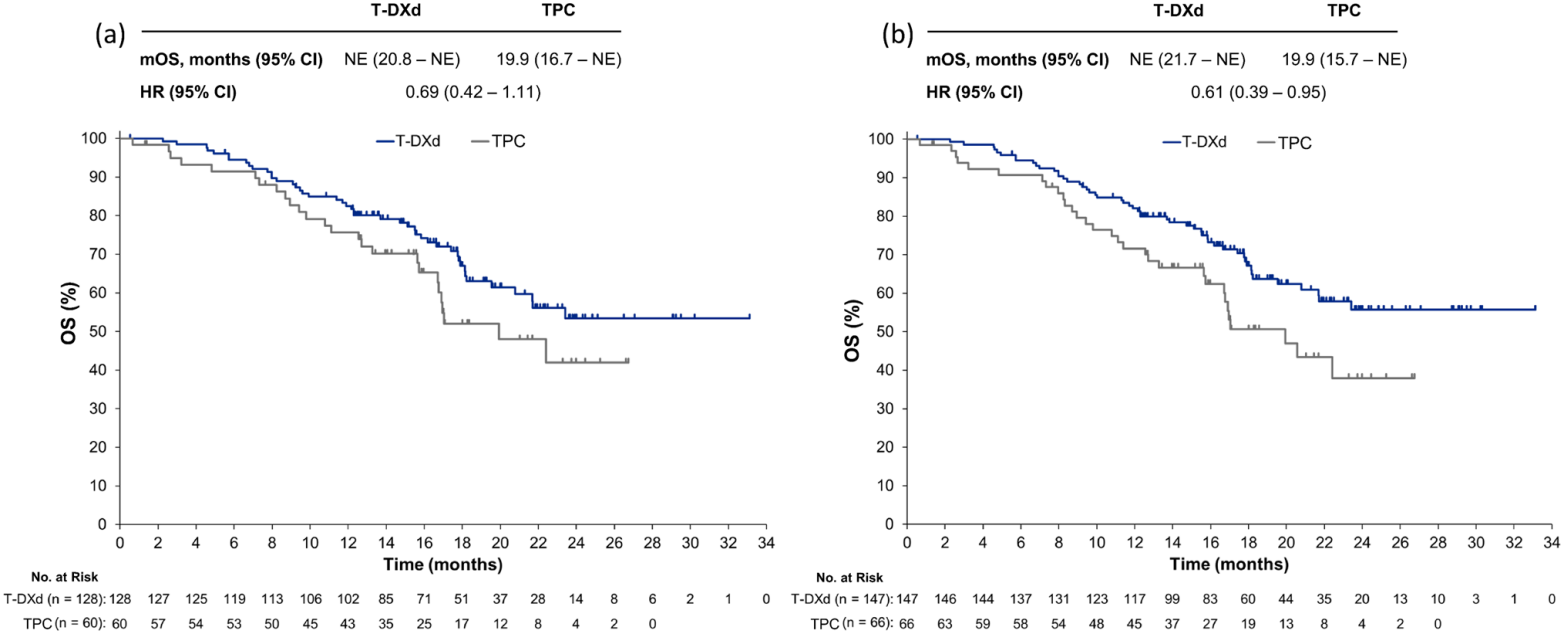
Overall survival in **a** the hormone receptor-positive Asian cohort, and **b** all Asian patients (hormone receptor-positive and -negative). *CI* confidence interval, *HR* hazard ratio, *mOS* median overall survival, *NE* not estimable, *T-DXd* trastuzumab deruxtecan, *TPC* treatment of physician’s choice

The median OS in the hormone receptor-positive cohort was not reached in the T-DXd group (95% CI 20.8 months–not estimable [NE]) and was 19.9 (16.7–NE) months in the TPC group (HR 0.69; 95% CI 0.42–1.11; Fig. 2a). In the overall population, the median OS was not reached in the T-DXd group (95% CI 21.7 months–NE) and was 19.9 (15.7–NE) months in the TPC group (HR 0.61; 95% CI 0.39–0.95; Fig. 2b).

In the overall Asian population, the confirmed ORR was better in the T-DXd group than in the TPC group (53.7% [95% CI 45.3–62.0%] vs 13.6% [95% CI 6.4–24.3%], respectively; Table 2). In the T-DXd group, nine patients (6.1%) had a CR, and 70 (47.6%) had a PR compared with one (1.5%) and eight (12.1%) patients, respectively, in the TPC group. Fifty-five patients (37.4%) had stable disease in the T-DXd group compared with 32 (48.5%) in the TPC group. The CBR was 73.5% (95% CI 65.6–80.4%) with T-DXd and 31.8% (95% CI 20.9–44.4%) with TPC, and the DCR was 91.2% (95% CI 85.4–95.2%) and 62.1% (95% CI 49.3–73.8%) in the respective groups.

**Table 2:** Overall efficacy in Asian patients included in the DESTINY-Breast04 study

	Hormone receptor-positive Asian patients		All Asian patients	
	T-DXd n = 128	TPC n = 60	T-DXd n = 147	TPC n = 66
PFS				
Median (95% CI), months	10.9 (8.4–14.7)	5.3 (4.2–6.8)	10.9 (9.0–13.8)	4.6 (2.8–6.4)
HR (95% CI) for disease progression or death	0.41 (0.28–0.58)		0.38 (0.27–0.53)	
OS				
Median (95% CI), months	NR (20.8–NE)	19.9 (16.7–NE)	NR (21.7–NE)	19.9 (15.7–NE)
HR (95% CI) for death	0.69 (0.42–1.11)		0.61 (0.39–0.95)	
Treatment response				
Best overall response, n (%)				
CR	8 (6.3)	1 (1.7)	9 (6.1)	1 (1.5)
PR	59 (46.1)	7 (11.7)	70 (47.6)	8 (12.1)
Stable disease	49 (38.3)	30 (50.0)	55 (37.4)	32 (48.5)
PD	8 (6.3)	17 (28.3)	8 (5.4)	20 (30.3)
Not evaluable	4 (3.1)	5 (8.3)	5 (3.4)	5 (7.6)
Confirmed ORR,^a^ n (%)	67 (52.3)	8 (13.3)	79 (53.7)	9 (13.6)
95% CI^b^	43.3–61.2	5.9–24.6	45.3–62.0	6.4–24.3
DCR^c^ n (%)	116 (90.6)	38 (63.3)	134 (91.2)	41 (62.1)
95% CI^b^	84.2–95.1	49.9–75.4	85.4–95.2	49.3–73.8
CBR^d^ n (%)	94 (73.4)	20 (33.3)	108 (73.5)	21 (31.8)
95% CI^b^	64.9–80.9	21.7–46.7	65.6–80.4	20.9–44.4
Median (range) time to first CR/PR response, months	2.8 (1.2–13.9)	2.3 (1.2–7.2)	2.7 (1.2–13.9)	2.8 (1.2–7.2)
Median (95% CI) duration of CR/PR response, months	11.9 (8.3–16.3)	6.6 (3.1–15.3)	11.9 (8.3–15.2)	6.6 (3.1–15.3)

*CBR* clinical benefit rate, *CI* confidence interval, *CR* complete response, *DCR* disease control rate, *HR* hazard ratio, *NE* not estimable, *NR* not reached, *ORR* objective response rate, *OS* overall survival, *PD* progressive disease, *PFS* progression free survival, *PR* partial response, *T-DXd* trastuzumab deruxtecan, *TPC* treatment of physician’s choice

^a^ORR= CR + PR

^b^Based on the Clopper-Pearson method for single proportion and for the difference of two proportions with continuity correction

^c^DCR = CR + PR + stable disease

^d^CBR = CR + PR +  > 6 months’ stable disease

In the overall Asian population, the median time to first CR or PR response was 2.7 (95% CI 1.2–13.9) months with T-DXd and 2.8 (95% CI 1.2–7.2) months with TPC (Table 2). The median duration of CR or PR response was 11.9 (95% CI 8.3–15.2) months and 6.6 (95% CI 3.1–15.3) months in the respective groups. Similar results were observed in the patients with hormone receptor-positive HER2-low mBC (Table 2).

### Safety

Safety was assessed in 147 patients in the T-DXd group and in 63 in the TPC group (Online Resource 2). The median treatment duration was 8.4 months with T-DXd and 3.5 months with TPC.

The most common drug-related TEAEs of any grade with T-DXd were nausea (76.2%), fatigue (45.6%), and alopecia (44.9%); the incidence of each of these events was lower in the TPC group (22.2%, 34.9%, and 34.9%, respectively; Table 3). In contrast, the most common drug-related TEAEs of any grade with TPC were hematologic disorders, such as neutropenia (73.0%) and leukopenia (52.4%), and transaminases increased (38.1%); the incidence of these events was lower in the T-DXd group (41.5%, 40.1%, and 34.7%, respectively).

**Table 3:** Drug-related treatment-emergent adverse events in Asian patients in the safety analysis set

System Organ Class Preferred term, n (%)	T-DXd n = 147		TPC n = 63	
	Any grade	Grade ≥ 3	Any grade	Grade ≥ 3
Blood and lymphatic system disorders				
Neutropenia^a^	61 (41.5)	24 (16.3)	46 (73.0)	38 (60.3)
Anemia^b^	61 (41.5)	19 (12.9)	16 (25.4)	4 (6.3)
Leukopenia^c^	59 (40.1)	17 (11.6)	33 (52.4)	21 (33.3)
Thrombocytopenia^d^	49 (33.3)	13 (8.8)	6 (9.5)	1 (1.6)
Metabolism and nutrition disorders				
Decreased appetite	62 (42.2)	6 (4.1)	18 (28.6)	1 (1.6)
Gastrointestinal disorders				
Nausea	112 (76.2)	8 (5.4)	14 (22.2)	0
Vomiting	52 (35.4)	0	5 (7.9)	0
Constipation	45 (30.6)	0	7 (11.1)	0
Skin and subcutaneous tissue disorders				
Alopecia	66 (44.9)	0	22 (34.9)	0
General disorders and administration site conditions				
Fatigue^e^	67 (45.6)	12 (8.2)	22 (34.9)	3 (4.8)
Investigations				
Transaminases increased^f^	51 (34.7)	4 (2.7)	24 (38.1)	10 (15.9)

*T-DXd* trastuzumab deruxtecan, *TPC* treatment of physician’s choice

^a^This category includes the preferred terms neutrophil count decreased and neutropenia

^b^This category includes the preferred terms hemoglobin decreased, red-cell count decreased, anemia, and hematocrit decreased

^c^This category includes the preferred terms white-cell count decreased and leukopenia

^d^This category includes the preferred terms platelet count decreased and thrombocytopenia

^e^This category includes the preferred terms fatigue, asthenia, and malaise

^f^This category includes the preferred terms aminotransferase levels increased, aspartate aminotransferase increased, alanine aminotransferase increased, γ-glutamyltransferase increased, liver function test abnormal, and hepatic function abnormal

The most common grade ≥ 3 drug-related TEAEs in either group were neutropenia (16.3% with T-DXd vs 60.3% with TPC) and leukopenia (11.6% vs 33.3%). The other most common grade ≥ 3 drug-related TEAEs were anemia with T-DXd (12.9% vs 6.3% with TPC) and transaminases increased with TPC (15.9% vs 2.7% with T-DXd; Table 3). Decreased left ventricular ejection fraction (LVEF) occurred in three patients (2.0%) in the T-DXd group; all three incidences were grade 2 in severity. No patients in the TPC group experienced decreased LVEF (data not shown).

In the overall population, adjudicated drug-related ILD or pneumonitis occurred in 21 patients (14.3%) in the T-DXd group; 12 events (8.2%) were grade 1 in severity, eight (5.4%) grade 2, and one (0.7%) grade 3; no events were grade 4 or 5 (Table 4). No adjudicated drug-related ILD or pneumonitis was reported in the TPC group. Patients from Japan and China represented the majority of those in whom adjudicated drug-related ILD or pneumonitis occurred (15 and five patients, respectively, of the 21 patients who experienced this TEAE of any grade; Table 4). Patients from Japan in the T-DXd group had the highest incidences of adjudicated drug-related ILD or pneumonitis (any grade: 26.8%; grade 1: 17.9%; and grade 2: 8.9%; Table 4). In the overall population, all events of adjudicated drug-related ILD or pneumonitis were low-grade (i.e., grade < 3 events), with the exception of one patient (2.2%) in the China subgroup who reported one grade 3 event (Table 4). The median time to the onset of the first adjudicated drug-related ILD with T-DXd was 168 (range 36–710) days in the overall population; it was also 168 days in those patients from China (range 125–255 days) and Japan (range 36–379 days). In the overall population, 11 out of 21 patients (52.4%) with adjudicated drug-related ILD in the T-DXd group had recovered from the event by the data cut-off.

**Table 4:** Adjudicated drug-related interstitial lung disease or pneumonitis in Asian patients in the safety analysis set

	Overall Asian population		Japan subgroup		China subgroup	
	T-DXd n = 147	TPC n = 63	T-DXd n = 56	TPC n = 29	T-DXd n = 45	TPC n = 17
Any grade	21 (14.3)	0	15 (26.8)	0	5 (11.1)	0
Grade 1	12 (8.2)	0	10 (17.9)	0	1 (2.2)	0
Grade 2	8 (5.4)	0	5 (8.9)	0	3 (6.7)	0
Grade 3	1 (0.7)	0	0	0	1 (2.2)	0
Grade 4	0	0	0	0	0	0
Grade 5	0	0	0	0	0	0

*T-DXd* trastuzumab deruxtecan, *TPC* treatment of physician’s choice

## Discussion

BC represents a significant healthcare and humanistic burden in Asian countries, with East Asia having the highest number of BC incidence cases in 2019 and the greatest increase in age-standardized incidence rate from 1990 to 2019 in the Global Burden of Disease study, and South Asia, East Asia, and Southeast Asia having the highest disability-adjusted life year burden associated with BC in 2019 [18]. Thus, more effective interventions are required for Asian patients with all types of BC, including advanced/metastatic disease.

This subgroup analysis showed that T-DXd provided clinically meaningful PFS and OS benefits over TPC in Asian patients with HER2-low mBC, including both hormone receptor-positive and hormone receptor-negative patients, with a similar safety profile in the two treatment groups. Compared with TPC, T-DXd was associated with a lower risk of disease progression or death among hormone receptor-positive patients (by 59%) and in the overall population (by 62%), as well as a 31% and 39% lower risk of death, respectively. In addition, T-DXd demonstrated a better antitumor response than TPC. These results are consistent with those observed in the overall DESTINY-Breast04 population, which demonstrated a significant and sustained PFS benefit with T-DXd versus TPC in patients with hormone receptor-positive disease after a median follow-up of 18.4 months (HR 0.51; p < 0.001), providing a 49.0% lower risk of disease progression or death with T-DXd versus TPC and consistent efficacy irrespective of HER2 IHC score [16].

Previous clinical studies have shown modest benefits with chemotherapy in heavily pretreated patients with mBC, including those who had received anthracycline or taxane therapy [19, 20]. In the global phase 3 EMBRACE trial, which assessed survival outcomes with eribulin versus TPC, median OS and PFS with eribulin was 13.1 and 3.7 months, respectively [19]. In the current study, median OS and PFS for the overall Asian population receiving TPC (i.e., the control arm; 19.9 and 4.6 months, respectively) were slightly longer than those reported with eribulin in EMBRACE. The longer median OS in the current subgroup analysis may be partly attributed to the different types of chemotherapy administered compared with EMBRACE (i.e., TPC [capecitabine, eribulin, gemcitabine, paclitaxel, or nab-paclitaxel] vs eribulin alone), the use of eribulin in an earlier line setting than in EMBRACE, and potential differences in the patient populations. The Pan-Asian adapted ESMO guidelines recommend a number of options for beyond second-line therapy for patients with mBC, including bevacizumab added to a taxane or capecitabine in those with hormone receptor-positive HER2-negative BC [11]. These guidelines also suggest that sacituzumab govitecan may represent a new treatment option for patients with advanced hormone receptor-positive HER2-negative BC that has progressed despite prior treatments (including endocrine therapy, cyclin-dependent kinase 4/6 inhibitors, and ≥ 2 lines of chemotherapy) [11], on the basis of the TROPiCS-02 study [21].

In this subgroup analysis in Asian patients, T-DXd had a similar safety profile to that observed in the overall DESTINY-Breast04 population [16] and in previous clinical trials of patients with HER2-positive mBC [22, 23]. In Asian patients, the most common drug-related TEAEs with T-DXd were nausea, fatigue, and alopecia, while hematologic disorders were more common with TPC. The most common grade ≥ 3 drug-related TEAE in both treatment groups in Asian patients was neutropenia, although the incidence of this event was higher with TPC (60.3% vs 16.3%).

ILD and pneumonitis have been identified as important risks with T-DXd [22, 23], requiring awareness, careful vigilance, and early intervention. In this subgroup analysis, the incidence of adjudicated drug-related ILD or pneumonitis in Asian patients was 14.3%; however, these were mostly low-grade events, with only one (0.7%) grade 3 event reported. The incidence of ILD or pneumonitis was similar to that reported in the overall DESTINY-Breast04 population (12.1%) [16] and other clinical studies (10.5–15.4%) [22–24]. Interestingly, the incidence of ILD or pneumonitis with T-DXd was higher in patients from Japan (26.8%) compared with all Asian patients and the overall DESTINY-Breast04 populations; no grade ≥ 3 events were observed in the Japan subpopulation. These findings are similar to previous reports of higher risk of ILD or pneumonitis with T-DXd treatment in Japanese patients [24], and may be linked to underlying biologic factors or Japanese-specific management/monitoring practices. Further, a real-world study of T-DXd use in Japan since its launch in 2020 reported that the most common ILD radiologic patterns were organizing pneumonia, hypersensitivity pneumonitis, and diffuse alveolar damage (DAD), with the DAD subtype being associated with poor prognosis [25]. Based on experience of gefitinib-induced ILD [26], healthcare providers in Japan have a high awareness of ILD and more experience in proactively detecting ILD, even at grade 1 stage (i.e., ILD manifesting only as abnormal high-resolution computed tomography) and intensively managing ILD.

The main limitations of this subgroup analysis include its descriptive nature, the limited number of patients with hormone receptor-negative HER2-low mBC, and the sole focus on the East Asian patient population, which may limit the generalizability of these findings, although it should be noted that the findings are consistent with those observed in the primary analysis from DESTINY-Breast04.

In conclusion, T-DXd demonstrated clinically meaningful PFS and OS benefits compared with TPC in Asian patients with HER2-low mBC in the DESTINY-Breast04 study, regardless of hormone receptor status. In addition, T-DXd was tolerable, and no new safety signals were observed in Asian patients. These results were consistent with the overall DESTINY-Breast04 population, suggesting that T-DXd is an appropriate treatment regimen in Asian patients with HER2-low mBC. The demonstrated efficacy of T-DXd outside of classically defined HER2-positive tumors, paired with international guidelines establishing T-DXd as a standard of care in HER2-low mBC populations, represents a major paradigm shift in anti-HER2 targeted therapy.

### Supplementary Information

Below is the link to the electronic supplementary material.

Supplementary file1 (DOCX 70 KB)

Supplementary file2 (DOCX 42 KB)

## Data Availability

Anonymized individual participant data (IPD) on completed studies and applicable supporting clinical study documents may be available upon request at https://vivli.org/. In cases where clinical study data and supporting documents are provided pursuant to our company policies and procedures, Daiichi Sankyo Companies will continue to protect the privacy of company and our clinical study subjects. Details on data sharing criteria and the procedure for requesting access can be found at this web address: https://vivli.org/ourmember/daiichi-sankyo/.

## Abbreviations

ADC: Antibody–drug conjugate
BC: Breast cancer
BICR: Blinded independent central review
CBR: Clinical benefit rate
CDK4/6: Cyclin-dependent kinase 4 and 6
CI: Confidence interval
CR: Complete response
DCR: Disease control rate
DAD: Diffuse alveolar damage
ECOG PS: Eastern Cooperative Oncology Group Performance Status
CrCl: creatinine clearance
ESMO: European Society for Medical Oncology
HER2: Human epidermal growth factor receptor 2
HR: Hazard ratio
IHC: Immunohistochemistry
ILD: Interstitial lung disease
ISH: In situ hybridization
LVEF: Left ventricular ejection fraction
mBC: Metastatic breast cancer
NE: Not estimable
ORR: Objective response rate
OS: Overall survival
PD: Progressive disease
PFS: Progression-free survival
PR: Partial response
TEAE: Treatment-emergent adverse event
T-DXd: Trastuzumab deruxtecan
TPC: Treatment of physician’s choice
