# Supplementary material for: Trastuzumab deruxtecan versus treatment of physician’s choice in previously treated Asian patients with HER2-low unresectable/metastatic breast cancer: subgroup analysis of the DESTINY-Breast04 study
Source: Breast Cancer. 2024 Jun 17;31(5):858–68. doi: 10.1007/s12282-024-01600-7 (PMC11341650; doi:10.1007/s12282-024-01600-7)
Supplement: Supplementary file 1 — Supplementary file1 (DOCX 70 KB) [file 12282_2024_1600_MOESM1_ESM.docx]

## Online Resource 1.

Disposition of Asian patients as of data cut-off (January 11, 2022).


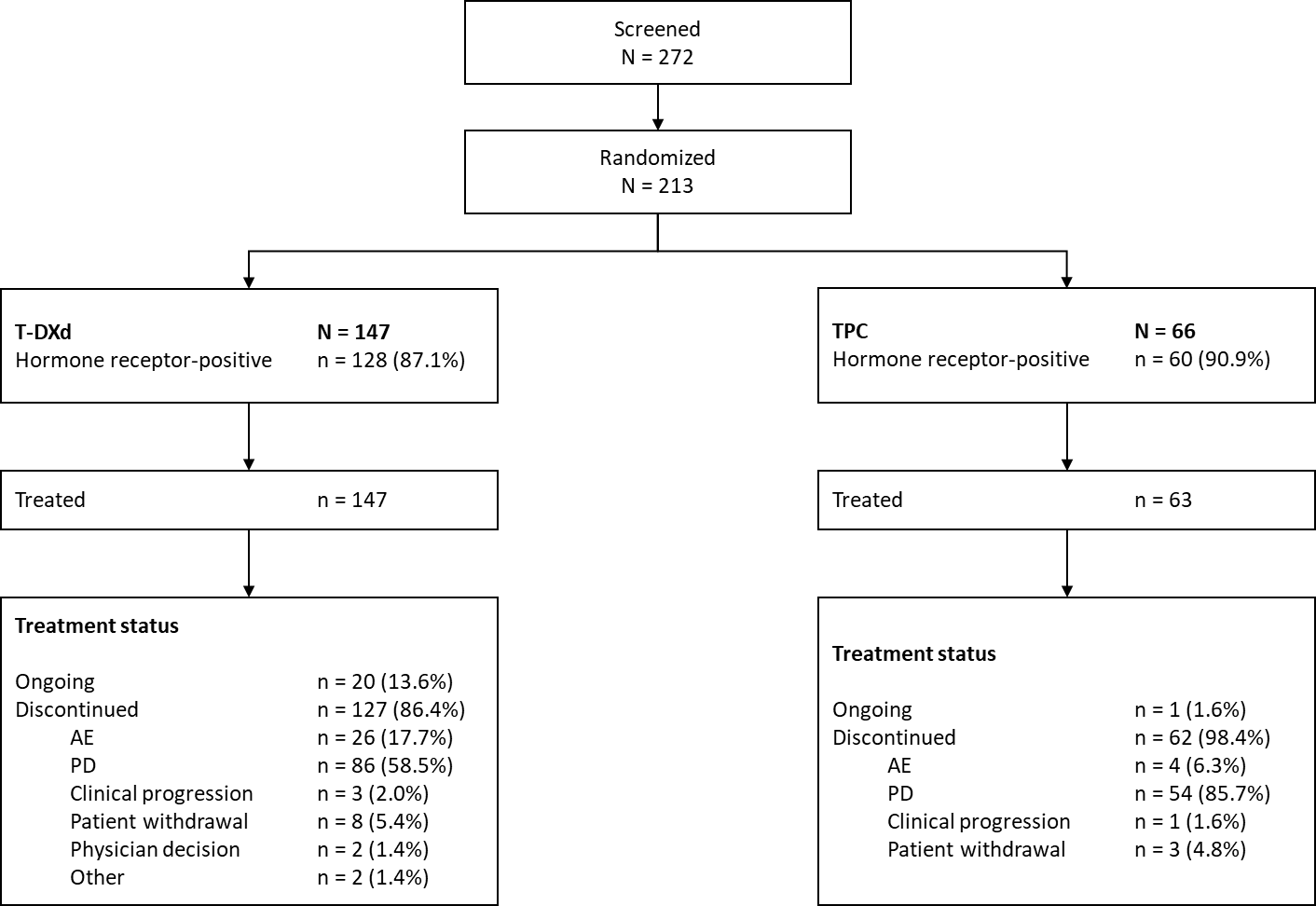


AE, adverse event; PD, progressive disease; T-DXd, trastuzumab deruxtecan; TPC, treatment of physician’s choice.
