# Supplementary material for: Trastuzumab deruxtecan versus treatment of physician’s choice in previously treated Asian patients with HER2-low unresectable/metastatic breast cancer: subgroup analysis of the DESTINY-Breast04 study
Source: Breast Cancer. 2024 Jun 17;31(5):858–68. doi: 10.1007/s12282-024-01600-7 (PMC11341650; doi:10.1007/s12282-024-01600-7)
Supplement: Supplementary file 2 — Supplementary file2 (DOCX 42 KB) [file 12282_2024_1600_MOESM2_ESM.docx]

## Online Resource 2.

Safety summary in Asian patients

|  | **T-DXd**  **n = 147** | **TPC**  **n = 63** |
| --- | --- | --- |
| Treatment duration, median (range), months | 8.4 (0.7, 33.3) | 3.5 (0.5, 17.5) |
| TEAEs, n (%) | 147 (100) | 63 (100) |
| Grade ≥3 TEAEs | 87 (59.2) | 48 (76.2) |
| Serious TEAEs | 38 (25.9) | 15 (23.8) |
| TEAEs associated with study drug discontinuation | 26 (17.7) | 4 (6.3) |
| TEAEs associated with study drug interruption | 60 (40.8) | 28 (44.4) |
| TEAEs associated with dose reduction | 42 (28.6) | 30 (47.6) |
| TEAEs associated with death | 2 (1.4) | 1 (1.6) |

T-DXd, trastuzumab deruxtecan; TEAE, treatment-emergent adverse event; TPC, treatment of physician’s choice.
